# The virtual reality classroom: a randomized control trial of medical student knowledge of postpartum hemorrhage emergency management

**DOI:** 10.3389/fmed.2024.1371075

**Published:** 2024-03-19

**Authors:** Kristyn Dunlop, Grace Dillon, Aoife McEvoy, Daniel Kane, Shane Higgins, Eleni Mangina, Fionnuala M. McAuliffe

**Affiliations:** ^1^UCD Perinatal Research Centre, University College Dublin, The National Maternity Hospital, Dublin, Ireland; ^2^Department of Obstetrics & Gynecology, Royal College of Surgeons in Ireland, Rotunda Hospital, Dublin, Ireland; ^3^School of Computer Science, University College Dublin, Dublin, Ireland

**Keywords:** medical education, obstetrics and gynecology, virtual reality learning environment, postpartum hemorrhage, balloon tamponade

## Abstract

**Objective:**

To investigate the use of a virtual reality learning environment (VRLE) to enhance medical student knowledge of postpartum hemorrhage (PPH) emergency management and insertion of a postpartum balloon.

**Methods:**

A randomized control trial involving medical students from University College Dublin, Ireland. Participants were randomly allocated to the intervention group (VRLE tutorial) or control group (PowerPoint tutorial on the same topic). All participants completed pre-learning experience and post-learning experience surveys. Both groups were timed and assessed on postpartum balloon insertion technique on a model pelvis. The primary outcome was assessment of student knowledge. Secondary outcomes included confidence levels, time taken to complete the task, technique assessment, satisfaction with the learning environment, and side effects of VR.

**Results:**

Both learning experiences significantly (*p* < 0.001) enhanced student performance on the post-learning experience multiple choice questionnaire, with no difference between the intervention and control groups. In the intervention group, time for task completion was significantly less compared to the control group (1–2 min vs. 2–3 min, *p* = 0.039). Both learning experiences significantly (*p* < 0.001) enhanced student confidence, with no significant difference between intervention and control groups. 100% of the students using the VRLE enjoyed the experience, and 82.4% were very likely to recommend use of VRLE in medical education. 94.1% of the students felt the VRLE was beneficial over didactic teaching.

**Conclusion:**

Receiving formal instruction, regardless of format, enhances students’ knowledge and confidence of the topic covered. Students who received instruction via the VRLE assembled the postpartum balloon faster than students who received didactic teaching. VR may be beneficial in teaching hands-on procedural skills in obstetrics and gynecology education.

## 1 Introduction

Postpartum hemorrhage (PPH) remains a leading cause of maternal mortality and morbidity worldwide (1). Prompt recognition and initiation of emergency management and resuscitation is crucial to reduce morbidity (1). The most common cause of PPH is uterine atony (1), and where uterotonic medications fail, balloon tamponade is a simple, effective, and potentially life-saving measure (2). Therefore, PPH emergency management is a critical concept in undergraduate obstetrics and gynecology teaching in order to prepare our future physicians for clinical practice.

Unfortunately, due to the sensitive nature of many aspects of clinical obstetrics and gynecology, medical students often find it difficult to get hands-on clinical experience in the specialty (3). Additionally, the centre in which students attend their clinical placements may have varying birth rates, making PPH a potentially rare event for students to encounter during the course of their clinical placement (4). During emergency situations, medical students are often silent observers, reducing their exposure to critical aspects of PPH emergency management (3). Therefore, simulated clinical environments are increasingly part of the medical school curriculum, in order to foster development of essential skills in prompt resuscitation, emergency management and communication in advance of clinical practice (5). Simulated clinical environments offer an opportunity to gain knowledge and confidence in emergency management skills, with the ultimate goal of preparing participants for prompt recognition and management of PPH, in order to improve patient outcomes (4). Additionally, simulated clinical environments provide students with an opportunity to practice hands-on skills which may not be commonly encountered (3), such as insertion of a postpartum balloon for uterine tamponade. These simulated clinical environments offer students a safe place to make mistakes, without risking patient safety in the clinical environment (4).

As technology has advanced and educational technology has become more accessible, virtual reality (VR) is increasingly being used to improve the educational experience (6). VR technology creates an immersive environment in which the participant can explore and manipulate multimedia sensory environments in real-time (7). VR has the potential to enhance the simulated clinical environment by creating a real-life multisensory clinical learning environment. Learning through simulation and VR utilises the constructivist educational theory, whereby learners construct knowledge through their interaction with the learning environment, rather than passively taking in information (8). VR learning tools are often self-directed, and promote active engagement of the student to navigate their own learning experience, thus resulting in the student forming their own knowledge through a self-regulated process (9). Additionally, VR has the benefit of supporting knowledge acquisition for procedural skills, where the user can repeat procedural steps hands-on through the VR headset as many times as required for the individual learner to feel confident with the procedure (10), facilitating practice without the risk of patient harm (11).

Therefore, the objective of our study was to assess the value of using VR in simulating training for medical students in management of PPH and insertion of a postpartum balloon for uterine tamponade. The topic of PPH was chosen as it is an emergency situation, and a critical concept to understand at the undergraduate level. Additionally, we were able to consolidate generalizable resuscitation skills, and introduce the procedural component of a postpartum balloon for uterine tamponade, which students’ would rarely see during their clinical placements. We hypothesized that VR would enhance the student learning experience and improve the insertion technique of the postpartum balloon, compared with traditional didactic teaching methods.

## 2 Materials and methods

A randomized control trial (RCT) of students in the clinical years of the undergraduate (6-year program) and graduate entry (4-year program) medical degree programs at University College Dublin (UCD) was conducted over 5 days from October 15–19, 2023. Graduate entry medical students are those who have completed a primary degree, and are now undertaking medicine as a second degree. Ethical approval for this study was obtained from the University College Dublin Research Ethics Committee.

### 2.1 Participants

Similar to previous successful studies conducted at the UCD Perinatal Research Centre, medical students were invited to take part in the study via class announcements on the university’s e-learning platform (12, 13), and announcements made during clinical placement. The announcements gave a brief overview of the study, without giving specific details of the content to be covered. Interested students contacted the study team via email, and an information leaflet and consent form was sent to each student for review. All students in the clinical years of study at UCD were eligible for participation, except those younger than 18 years of age, or with a medical condition including cardiac (e.g., pacemakers), binocular vision abnormalities, psychiatric disorders or epilepsy. Informed written consent from all participants was obtained on arrival to the teaching space. Participants were advised they could withdraw consent to participate at any time.

### 2.2 Randomization

**Figure 1:**
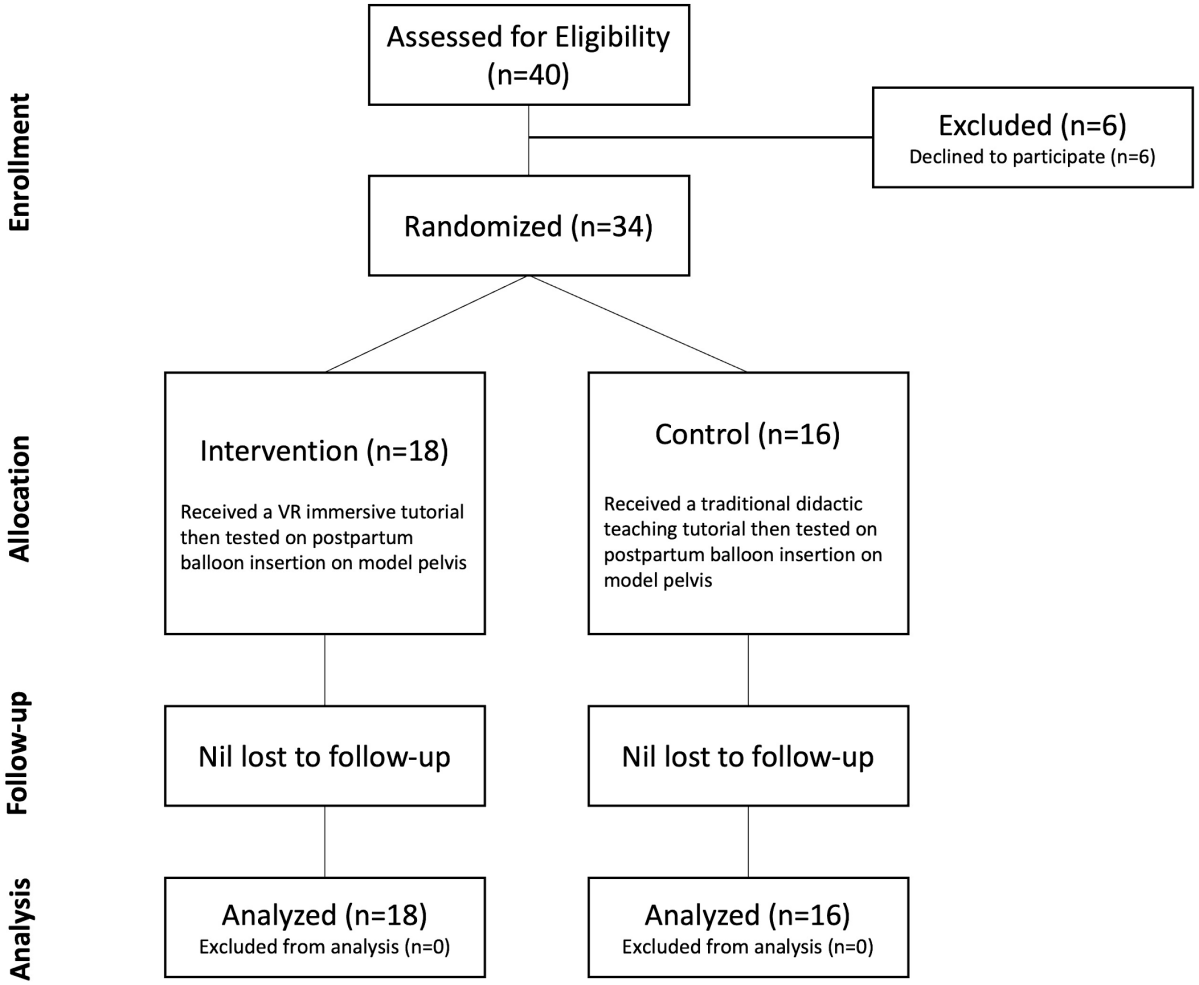
CONSORT (Consolidated Standards of Reporting Trials) diagram depicting the randomization and allocation process. VR – virtual reality.

Figure 1 depicts the randomization and allocation process for the study. Participants were randomly allocated to intervention or control groups through the use of sequentially numbered, opaque, sealed envelopes. The researcher conducting the study was not blinded to the group allocation given the nature of the study design, however, allocation was prospectively concealed to the students.

### 2.3 Intervention and control groups

**Figure 2:**
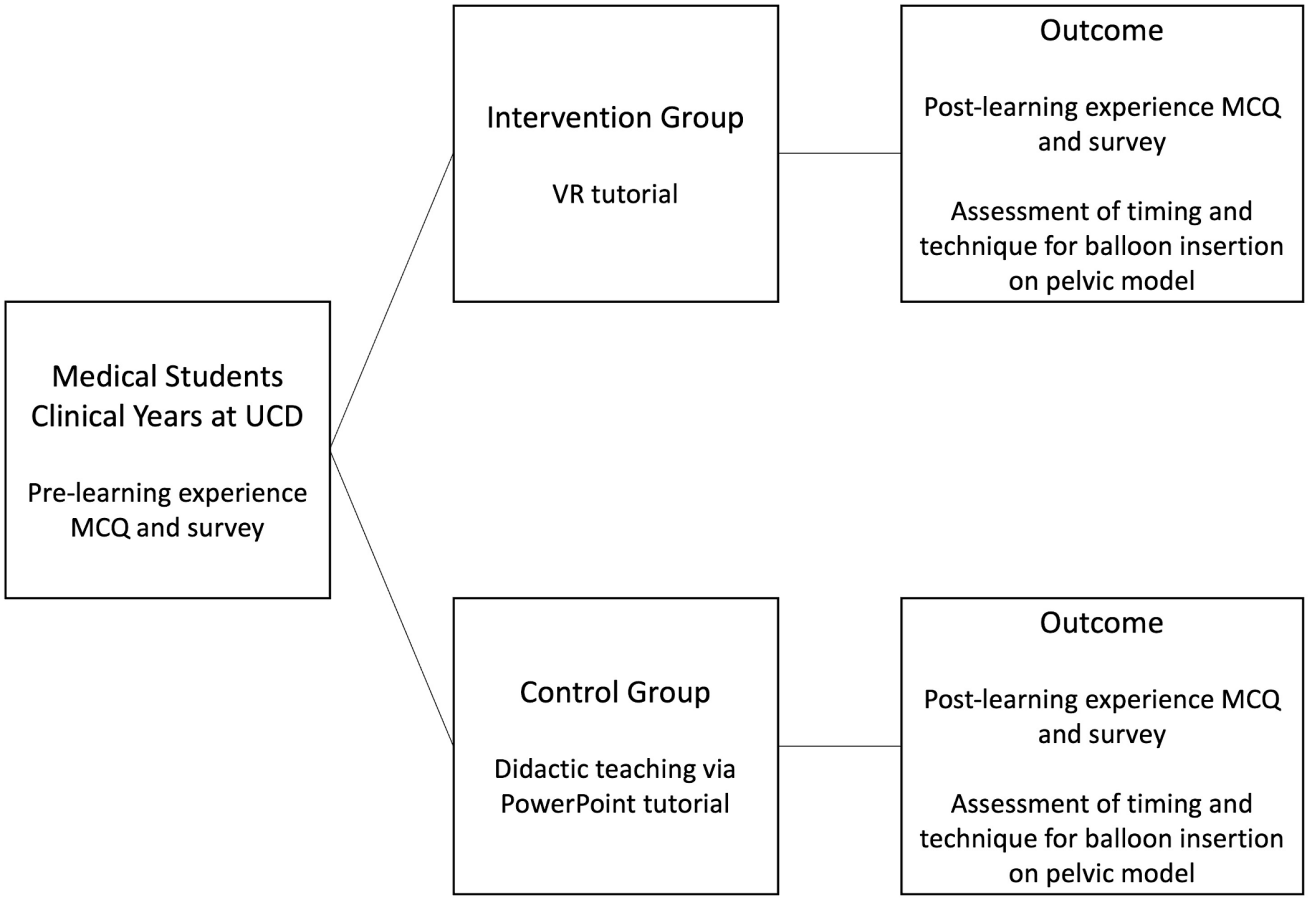
Study design. Pictorial representation of the study design and outcomes. UCD – University College Dublin, MCQ – multiple choice questionnaire, VR – virtual reality.

The intervention learning experience involved an immersive VR tutorial lasting for fifteen minutes, using an Oculus Lens-2 VR head-mounted display, designed to teach background material regarding PPH management and step-wise procedural instruction of postpartum balloon insertion. The VR learning environment (VRLE) was designed in collaboration with the UCD School of Computer Science and Magos, a company based in Athens, Greece, specializing in creation of VR programmes. Students assigned to the intervention group used the Oculus Headsets to participate in an interactive tutorial on PPH background, emergency management, and postpartum balloon insertion (Figure 2). Participants then carried out postpartum balloon insertion on a model pelvis, and were marked on insertion technique and time taken to complete insertion. All participants completed pre-learning experience and post-learning experience questionnaires, including multiple-choice questions (MCQ) regarding PPH knowledge and management, prior experience of PPH and VR, satisfaction with the learning experience, and attitudes towards use of VR in medical education.

The control group underwent a traditional didactic learning experience consisting of PowerPoint presentation (Figure 2). The factual content of the traditional tutorial replicated the VRLE tutorial. The traditional tutorial was conducted by the same clinical tutor for the purpose of consistency in the teaching, and lasted for fifteen minutes. Once students had completed the didactic learning experience, they were asked to carry out postpartum balloon insertion on a model pelvis, and were marked on insertion technique and time taken to complete insertion. All participants completed pre-learning experience and post-learning experience questionnaires, including multiple-choice questions regarding PPH knowledge and management, prior experience of PPH and VR, satisfaction with the learning experience and attitudes towards the use of VR in medical education.

### 2.4 Outcomes

The primary outcome was student knowledge regarding PPH management, as measured by MCQ scores pre-learning experience compared to MCQ scores post-learning experience. Pre-specified secondary outcomes included time taken to complete the task, technique for postpartum balloon insertion, improvement in confidence levels, virtual reality-side effects and satisfaction with the learning experience. Each participant underwent objective, timed assessment of balloon insertion technique on the model pelvis to assess if there was a difference for either learning experience. Confidence levels of managing PPH and postpartum balloon insertion were assessed pre- and post-learning experience. The intervention group was asked to complete a questionnaire regarding side-effects experienced while using the VR headsets. All students were asked a series of questions regarding their attitudes towards the use of VR in medical education and satisfaction with the learning experience.

### 2.5 Statistical analysis

Data collected was entered and coded into Microsoft Excel. Statistical analysis was performed using SPSS software package (version 27; SPSS, Chicago, Illinois). Descriptive statistics were calculated as frequency and percentage for categorical data or mean and standard deviation for normally distributed variables. The paired t-test was used to compare mean scores for the MCQ and confidence levels pre- and post-learning experience. Independent t-test was used to compare the technique assessment between the intervention and control groups. Chi-square test of independence was used to assess the difference between categorical variables, including timing of insertion of the postpartum balloon on the model pelvis, as the timings were grouped categorically in increments of one minute on the assessment survey. Statistical significance was defined as a *p*-value <0.05.

## 3 Results

A total of 40 medical students expressed interest in participating in the study, and were screened for eligibility – 6 students ultimately declined to participate. 34 students were randomly allocated, 18 to the intervention (VRLE) group, and 16 to the control group receiving didactic teaching only (Figure 1). There were no participants lost to follow up and none excluded from the analysis.

Table 1 outlines the background demographics of the study participants. In the total cohort, 25 participants (73.5%) were female, and 9 participants (26.5%) were male. The majority of participants (52.9%) were between the ages of 18 and 24 years, with 38.2% of participants between the ages of 25 and 34 years. 19 participants were from the undergraduate medicine course, of which 13 (38.2% of the total cohort) were in the final year of the program. 15 participants were from the graduate medicine course, of which 13 (38.2% of the total cohort) were in the final year of the program. 61.8% of the total cohort had no prior experience using VR at the time of the study, with only 1 participant having used VR 15–20 times in the past. In terms of familiarity with PPH, 58.8% of the participants had no prior knowledge or experience of PPH, and 41.2% of the participants had only theoretical knowledge of PPH. Similar to the demographics of the overall cohort, there were significantly (*p* = 0.038) more females in the intervention group (Table 1). The remaining participant demographics were not significantly different between intervention and control groups.

**Table 1:** Baseline demographics of participants in intervention and control groups.

	Total cohort (*n* = 34)	Control (*n* = 16)	Intervention (*n* = 18)	*p*-value
Sex				0.038*
Male	9 (26.5%)	7 (43.8%)	2 (11.1%)	
Female	25 (73.5%)	9 (56.3%)	16 (88.9%)	
Age				0.772
18–24	18 (52.9%)	8 (50%)	10 (55.6%)	
25–34	13 (38.2%)	7 (43.8%)	6 (33.3%)	
35–45	3 (8.8%)	1 (6.3%)	2 (11.1%)	
Undergraduate entry medical students				0.221
Year 4	1 (2.9%)	-	1 (5.6%)	
Year 5	5 (14.7%)	2 (12.5%)	3 (16.7%)	
Year 6	13 (38.2%)	4 (25%)	9 (50%)	
Graduate entry medical students				0.221
Year 3	2 (5.9%)	2 (12.5%)	–	
Year 4	13 (38.2%)	8 (50%)	5 (27.8%)	
Any prior experience using VR?				0.934
Yes	13 (38.2%)	7 (38.9%)	6 (37.5%)	
No	21 (61.8%)	11 (61.1%)	10 (62.5%)	
Number of times VR used in the past:				0.302
0–5	33 (97.1%)	7 (100%)	5 (31.2%)	
5–10	–	–	–	
15–20	1 (2.9%)	–	1 (6.2%)	
Prior experience in management of a PPH/insertion of an intrauterine balloon:				0.774
No prior knowledge or experience	20 (58.8%)	11 (61.1%)	9 (56.3%)	
Theoretical knowledge only	14 (41.2%)	7 (38.9%)	7 (43.8%)	
Theoretical knowledge and some clinical experience	–	–	–	
Regular management of PPH but never seen insertion of postpartum balloon	–	–	–	
Regular management of PPH, seen insertion of postpartum balloon, but not confident	–	–	–	
Regular management of PPH, confident or have inserted postpartum balloon	–	–	–	

There were significantly more female participants in the overall cohort, with more females completing the intervention. The remaining baseline demographics were not significantly different between the two groups. Number of participants (*n*) and percentage of overall group size are reported. Differences between groups were assessed using Chi-square test of independence. A *p*-value < 0.05 was considered statistically significant.VR – virtual reality, PPH – postpartum hemorrhage. Graduate entry medical students are those who have completed a primary degree, and are now undertaking medicine as a second degree.

With regards to the primary outcome testing student knowledge of PPH management, there was no significant difference in the mean MCQ score between the intervention or control groups when assessed before and after the learning experiences (Table 2). We noted a significant (*p* < 0.001) increase in mean MCQ score in both the intervention and control groups when comparing the pre- and post-learning experience MCQ scores (Table 3).

**Table 2:** MCQ scores for intervention and control groups.

	*n*	MCQ mark Mean (SD)	Mean difference (95% CI)	*p*-value
Pre-learning experience				
Control	16	6.7 (2.0)	0.8 (−0.2, 1.9)	0.113
Intervention	18	7.7 (0.8)		
Post-learning experience				
Control	16	9.3 (1.1)	−0.1 (−0.8, 0.6)	0.770
Intervention	18	9.4 (0.9)		

MCQs were scored out of 10 points. Mean scores (standard deviation) are presented. MCQ scores were assessed using independent *t*-tests to evaluate mean score differences between control and intervention groups pre-learning experience and post-learning experience.MCQ – multiple choice questionnaire, SD – standard deviation, CI – confidence interval.

**Table 3:** MCQ scores compared across time points.

	*n*	Pre-learning experience MCQ score Mean (SD)	Post-learning experience MCQ score Mean (SD)	Mean difference (95% CI)	*p*-value
Control	16	6.7 (2.0)	9.4 (0.9)	2.6 (1.6, 3.6)	<0.001
Intervention	18	7.7 (0.8)	9.3 (1.1)	1.6 (1.1, 2.1)	<0.001

MCQs were scored out of 10 points. Mean scores (standard deviation) are presented. MCQ scores were assessed using paired t-tests to evaluate the difference between the pre-learning and post-learning scores in each group. A *p*-value < 0.05 was considered statistically significant.MCQ – multiple choice questionnaire, SD – standard deviation, CI – confidence interval.

In terms of secondary outcomes, students were assessed on their ability to correctly assemble the components of a postpartum balloon and time taken to do so on a model pelvis. With regards the technique assessment, there was no difference in the mean score obtained between intervention or control groups (Table 4). We noted that students who participated in the intervention VRLE group assembled the postpartum balloon significantly (*p* = 0.039) faster [1.01–2 min vs. 2.01–3 min], compared with the control group (Table 4).

**Table 4:** Student assessment post-learning experience.

	Control (*n* = 16)	Intervention (*n* = 18)	Mean difference (95% CI)	*p*-value
Technique assessment				
Mean score (SD)	7.5 (0.8)	7.6 (0.7)	0.09 (−0.5, 0.6)	0.743
Timing assessment				
Time to completion (minutes)	2.01–3.0	1.01–2.0		0.039*

The technique assessment was scored out of 10 based on manufacturer’s guidelines. The timing assessment was marked categorically in 1 min increments. The technique assessment between groups was examined using independent *t*-tests. The timing assessment was evaluated using chi-square test of independence. A *p*-value < 0.05 was considered statistically significant.SD – standard deviation, CI – confidence interval.

With regards to confidence levels, there was no significant difference in the mean confidence level between the intervention or control group when assessed before and after the learning experiences (Table 5). We noted a significant (*p* < 0.001) improvement in mean confidence level following the learning experience, in both the intervention and control groups (Table 6). Ultimately, 33 out of 34 participants (97%) felt that their overall confidence improved following training in PPH management and postpartum balloon insertion.

**Table 5:** Confidence levels for intervention and control groups.

Group	*n*	Confidence level Mean (SD)	Mean difference (95% CI)	*p*-value
Pre-learning experience				
Control	16	1.1 (0.5)	0.2 (−0.2, 0.5)	0.417
Intervention	18	1.3 (0.6)		
Post-learning experience				
Control	16	2.7 (0.7)	0.1 (−0.4, 0.6)	0.639
Intervention	18	2.8 (0.7)		

Confidence levels were assessed using Likert-type rating scales, with 1 representing no confidence and 5 as maximum score. Mean scores (standard deviation) are presented. Confidence levels were examined using independent *t*-tests to assess mean score differences between groups pre-learning experience and post-learning experience.SD – standard deviation, CI – confidence interval.

**Table 6:** Confidence levels compared across time points.

Group	*n*	Pre-learning experience confidence level Mean (SD)	Post-learning experience confidence level Mean (SD)	Mean difference (95% CI)	*p*-value
Control	16	1.1 (0.5)	2.7 (0.7)	1.5 (1.2, 1.9)	<0.001
Intervention	18	1.3 (0.6)	2.8 (0.7)	1.5 (1.1, 1.8)	<0.001

Confidence levels were assessed using Likert-type rating scales, with 1 representing no confidence and 5 as maximum score. Mean scores (standard deviation) are presented. Confidence levels were examined using paired t-tests to examine the difference between the pre-learning and post-learning scores in each group. A *p*-value < 0.05 was considered statistically significant.SD – standard deviation, CI – confidence interval.

Table 7 describes the side effects experienced by students during the VRLE. 44.4% of those who used the VRLE experienced no side effects. Of those who did experience side effects, the most commonly reported symptoms were eye strain (27.8%), disorientation (27.8%), blurred/altered or double vision (16.7%), and nausea (16.7%).

**Table 7:** Side effects of VR.

Side-effect	*n* (%)
No side-effects	8 (44.4%)
Any side effect	10 (55.6%)
Dizziness	1 (5.6%)
Headache	1 (5.6%)
Blurred/altered or double vision	3 (16.7%)
Loss of awareness	–
Eye strain	5 (27.8%)
Eye or muscle twitching	–
Involuntary moving	–
Disorientation	5 (27.8%)
Impaired balance	–
Impaired hand-eye coordination	–
Excessive sweating	–
Increased salivation	–
Nausea	3 (16.7%)
Light-headedness	–
Discomfort or pain in the head or eyes	2 (11.1%)
Drowsiness	–
Fatigue	1 (5.6%)
Any symptoms similar to motion sickness	–

Side effects experienced by students while using the virtual reality learning environment. Participants completed a questionnaire regarding side effects experienced during the VRLE. Group (*n* = 18) and percentage of intervention group are reported for each side effect.

Table 8 describes student satisfaction with elements common to both learning experiences. Overall, students were highly satisfied with both learning experiences, with mean ratings for each domain ranging from “agree” to “strongly agree” on the Likert-style scale. There was no difference in student satisfaction between the intervention and control group.

**Table 8:** Student satisfaction of the learning experiences.

	Control (*n* = 16) Mean (SD)	Intervention (*n* = 18) Mean (SD)	*p*-value
Easy to use/follow	2.1 (0.7)	2.1 (0.8)	0.871
Clear purpose and objectives	1.4 (0.5)	1.6 (0.6)	0.293
Support during the learning experience	1.4 (0.6)	1.6 (0.9)	0.433
Designed for my specific level of knowledge and skills	1.6 (0.6)	1.9 (0.6)	0.182
Opportunity to enhance understanding	1.3 (0.5)	1.2 (0.4)	0.491
Learning benefitted from the learning experience	1.5 (0.6)	1.4 (0.6)	0.723
Opportunity for feedback	1.7 (0.6)	1.8 (0.9)	0.534
The learning experience was very realistic	1.9 (0.7)	2.2 (1.3)	0.324
Recommendation of the learning experience	1.3 (0.5)	1.4 (0.8)	0.812

Students were asked to rate their learning experience on a Likert-style scale of 1 = strongly agree to 5 = strongly disagree, for the above domains. Scores are presented as mean (standard deviation), and comparisons between control and intervention groups was done using independent *t*-tests. No significant differences were noted. A *p*-value < 0.05 was considered statistically significant.

In relation to the students’ views regarding the use of VR in medical education, 91.8% of participants agreed that VR technology could be useful as a learning tool in teaching obstetrics and gynecology topics to medical students. Of those who experienced the VRLE, 82.4% of participants were very likely to recommend its use in university and training in obstetrics and gynecology. 94.1% of the participants felt that the VRLE was beneficial over didactic teaching, and 100% of the participants who experienced the VRLE enjoyed the learning experience.

## 4 Discussion

### 4.1 Main findings

The objective of our randomized control trial was to pilot the use of a VRLE to enhance medical students’ knowledge of PPH management and insertion of a postpartum balloon for uterine tamponade. Receiving formal instruction, regardless of format, enhanced students’ knowledge of PPH emergency management. Both learning experiences (VRLE and didactic teaching session) enhanced student performance on the post-learning experience MCQ. Additionally, students who participated in the VRLE were quicker at insertion of the postpartum balloon insertion on the model pelvis. Both learning experiences (VRLE and didactic teaching session) also improved student confidence with PPH management, as assessed on the post-learning experience survey. While 55.6% of VRLE participants did experience side-effects of VR, none of them were significant enough to cease the learning experience.

### 4.2 Comparison with existing literature

Similar to previous studies examining the use of VR in embryology, and anatomy, including fetal and pelvic anatomy (12, 14, 15), no significant differences were demonstrated in the post-learning experience knowledge scores when VRLE was compared to didactic teaching. Previous research has demonstrated that learners who use a VRLE complete tasks significantly quicker than those who used other forms of learning (12, 16). We also found that our students who used the VRLE completed the assessment on the model pelvis quicker than the control group.

Simulation training in obstetrics and gynecology has demonstrated positive effects on the knowledge and skills obtained by the participants and improves overall satisfaction and self-confidence with the content taught through simulation (3). Similar to simulation training, our results suggest that a VRLE has the potential to enhance the knowledge and skills of the students and improve their self-rated confidence levels with the material. It has been suggested that VR may function as an alternative to simulated clinical environments (17).

Medical students find immersive technology, such as VR, very enjoyable to use and adds to their overall learning experience (12, 13, 18). Previous research has demonstrated that students who are motivated and engaged by novel learning methods are more likely to retain knowledge, and may have improved learning outcomes (19). Similarly, we found high levels of satisfaction among students who used the VRLE, with 100% of the students enjoying the experience and 82.4% very likely to recommend the use of VRLE in medical education in the future.

### 4.3 Implications for the future

Through this RCT, we have highlighted that a VRLE is an acceptable format in which to promote student engagement with concepts in obstetrics and gynecology. It is important to note that medical students are adult learners, and adults are more independent in their learning processes, striving for higher levels of autonomy and self-directed learning (20). In order to support this drive for self-directed learning, intrinsic motivation is required, which can be achieved when the student believes that the learning outcome is relevant to their practice (21). Therefore, the use of a VRLE specifically designed to achieve relevant learning outcomes may be beneficial to enhance student engagement and knowledge retention.

Additionally, VR has the potential to play a key role in creating a learning environment for acquisition of clinical skills in a safe environment (7). Medical education relies heavily on continued practice of procedural skills necessary to begin a clinical career (7). However, in obstetrics and gynecology, this can be very difficult for medical students to achieve due to the sensitive and occasionally invasive nature of the specialty (3). Additionally, medical students require increased exposure to emergency situations in order to prepare them for their clinical practice in the future (3). Therefore, VR is uniquely positioned to create an immersive simulated clinical environment in which students can practice procedural skills as many times as necessary to enhance preparedness for their future clinical careers.

### 4.4 Strengths and Limitations

A strength of this study is that it is a randomized control trial assessing the use of a VRLE in enhancing medical student knowledge of an important emergency management topic, and assessing its use for teaching rarely encountered procedural skills. A limitation of the current study is the small sample size, which may have led us to be under-powered to discover differences in knowledge acquisition. Additionally, as in previous studies (12, 13, 15), side-effects due to cybersickness are acknowledged as a limitation to the use of VR technology. However, with advancement of VR technology, and repeated exposure to the technology, side effects are expected to decrease (22). Additionally, the cost of VR is acknowledged as a potential barrier to widespread use. Thus far, VR in medical education has not been widely available due to high costs and lack of evidence of its efficacy (23). Once the VRLE has been developed, it can be used repeatedly without further development costs, however it is acknowledged that regular maintenance and software updates are required to ensure function of the VR headsets. Of note, recent technological advancements have reduced the cost of VR headsets, making them more affordable (24). Future studies with larger cohorts of medical students could further explore the use of the VRLE as learning tools for medical students, especially for procedural skills.

## 5 Conclusion

Receiving formal instruction, regardless of format, enhances students’ knowledge and confidence of the topic covered. Students who received instruction via the VRLE assembled the postpartum balloon faster than students who received didactic teaching. Use of VRLE in medical education represents an acceptable learning environment in which to engage students in order to enhance the overall learning experience. VR may be beneficial in teaching hands-on procedural skills in obstetrics and gynecology education.

## Data availability statement

The raw data supporting the conclusions of this article will be made available by the authors, without undue reservation.

## Ethics statement

The studies involving humans were approved by University College Dublin Research Ethics Committee. The studies were conducted in accordance with the local legislation and institutional requirements. The participants provided their written informed consent to participate in this study.

## Author contributions

KD: Data curation, Formal analysis, Investigation, Methodology, Writing – original draft, Writing – review & editing, Project administration. GD: Data curation, Writing – original draft, Writing – review & editing, Formal analysis. AM: Conceptualization, Methodology, Project administration, Writing – review & editing. DK: Conceptualization, Software, Writing – review & editing, Methodology. SH: Funding acquisition, Supervision, Writing – review & editing, Conceptualization, Validation. EM: Software, Supervision, Writing – review & editing, Validation. FM: Conceptualization, Funding acquisition, Methodology, Supervision, Writing – review & editing, Project administration, Validation.

## References

[1] Mavrides EAS Chandraharan E Collins P Green L Hunt BJ Riris S. Prevention and Management of Postpartum Haemorrhage: Green-top guideline no. 52. BJOG. (2017) 124:e106–49. doi: 10.1111/1471-0528.14178 27981719

[2] Purandare CN Nazareth AK Ryan G Purandare NC. Role of balloon tamponade as a therapeutic non-surgical tool in controlling obstetric and gynecological hemorrhage in low-resource countries. J Obstet Gynaecol India. (2022) 72:285–90. doi: 10.1007/s13224-022-01662-7, PMID: 35923509 PMC9339450

[3] Tauscher A Stepan H Todorow H Rotzoll D. Interteam PERINAT—interprofessional team collaboration in undergraduate midwifery and medical education in the context of obstetric emergencies: presentation of simulation scenarios and empirical evaluation results. GMS J Med Educ. (2023) 40:Doc20. doi: 10.3205/zma001602 37361251 PMC10285368

[4] Minehart RD Gallin H. Postpartum hemorrhage: the role of simulation. Best Pract Res Clin Anaesthesiol. (2022) 36:433–9. doi: 10.1016/j.bpa.2022.11.002, PMID: 36513437

[5] Shore EM Davidson A Arnason M Kara H Shah A Shah R. Bridging the gap: incorporating simulation into obstetrics and Gynaecology undergraduate medical education. J Obstet Gynaecol Can. (2019) 41:191–196.e2. doi: 10.1016/j.jogc.2018.03.016, PMID: 30316714

[6] Jin J Bridges SM. Educational technologies in problem-based learning in health sciences education: a systematic review. J Med Internet Res. (2014) 16:e251. doi: 10.2196/jmir.3240, PMID: 25498126 PMC4275485

[7] Ryan GV Callaghan S Rafferty A Higgins MF Mangina E McAuliffe F. Learning outcomes of immersive Technologies in Health Care Student Education: systematic review of the literature. J Med Internet Res. (2022) 24:e30082. doi: 10.2196/30082, PMID: 35103607 PMC8848248

[8] McGaghie WC Harris IB. Learning theory foundations of simulation-based mastery learning. Simul Healthc. (2018) 13:S15–20. doi: 10.1097/SIH.0000000000000279 29373384

[9] Taylor DC Hamdy H. Adult learning theories: implications for learning and teaching in medical education: AMEE guide no. 83. Med Teach. (2013) 35:e1561–72. doi: 10.3109/0142159X.2013.828153, PMID: 24004029

[10] Izard SG Juanes JA Garcia Penalvo FJ Estella JMG Ledesma MJS Ruisoto P. Virtual reality as an educational and training tool for medicine. J Med Syst. (2018) 42:50. doi: 10.1007/s10916-018-0900-2 29392522

[11] Graafland M Schraagen JM Schijven MP. Systematic review of serious games for medical education and surgical skills training. Br J Surg. (2012) 99:1322–30. doi: 10.1002/bjs.8819, PMID: 22961509

[12] Kane D Ryan G Mangina E McAuliffe FM. A randomized control trial of a virtual reality learning environment in obstetric medical student teaching. Int J Med Inform. (2022) 168:104899. doi: 10.1016/j.ijmedinf.2022.104899, PMID: 36335797

[13] Ryan G Rafferty A Murphy J Higgins MF Mangina E McAuliffe FM. Virtual reality learning: a randomized controlled trial assessing medical student knowledge of fetal development. Int J Gynaecol Obstet. (2023) 162:292–9. doi: 10.1002/ijgo.14684, PMID: 36883288

[14] Arents V de Groot PCM Struben VMD van Stralen KJ. Use of 360 degrees virtual reality video in medical obstetrical education: a quasi-experimental design. BMC Med Educ. (2021) 21:202. doi: 10.1186/s12909-021-02628-5, PMID: 33836736 PMC8035054

[15] Ryan G Callaghan S Rafferty A Murphy J Higgins M Barry T. Virtual reality in midwifery education: a mixed methods study to assess learning and understanding. Nurse Educ Today. (2022) 119:105573. doi: 10.1016/j.nedt.2022.105573, PMID: 36206631

[16] Nickel F Brzoska JA Gondan M Rangnick HM Chu J Kenngott HG. Virtual reality training versus blended learning of laparoscopic cholecystectomy: a randomized controlled trial with laparoscopic novices. Medicine (Baltimore). (2015) 94:e764. doi: 10.1097/MD.0000000000000764, PMID: 25997044 PMC4602875

[17] Birkham SL Calogiuri G Martinsen R. Advancing immersive virtual reality-based simulation practices: developing an evidence-based and theory-driven pedagogical framework for VR-based simulations of non-technical skills among healthcare professionals. Interact Learn Environ. (2023):1–13. doi: 10.1080/10494820.2023.2186896

[18] Guetterman TC Sakakibara R Baireddy S Kron FW Scerbo MW Cleary JF. Medical Students' experiences and outcomes using a virtual human simulation to improve communication skills: mixed methods study. J Med Internet Res. (2019) 21:e15459. doi: 10.2196/15459, PMID: 31774400 PMC6906619

[19] Huang H-M Rauch U Liaw S-S. Investigating learners’ attitudes toward virtual reality learning environments: based on a constructivist approach. Comput Educ. (2010) 55:1171–82. doi: 10.1016/j.compedu.2010.05.014

[20] Zigmont JJ Kappus LJ Sudikoff SN. Theoretical foundations of learning through simulation. Semin Perinatol. (2011) 35:47–51. doi: 10.1053/j.semperi.2011.01.002 21440810

[21] Ryan RM Deci EL. Self-determination theory and the facilitation of intrinsic motivation, social development, and well-being. Am Psychol. (2000) 55:68–78. doi: 10.1037/0003-066X.55.1.68, PMID: 11392867

[22] Kim J Luu W Palmisano S. Multisensory integration and the experience of scene instability, presence and cybersickness in virtual environments. Comput Hum Behav. (2020) 113:106484:106484. doi: 10.1016/j.chb.2020.106484

[23] Farra SL Gneuhs M Hodgson E Kawosa B Miller ET Simon A. Comparative cost of virtual reality training and live exercises for training Hospital Workers for Evacuation. Comput Inform Nurs. (2019) 37:446–54. doi: 10.1097/CIN.0000000000000540, PMID: 31166203 PMC7231540

[24] Hamilton D McKechnie J Edgerton E Wilson C. Immersive virtual reality as a pedagogical tool in education: a systematic literature review of quantitative learning outcomes and experimental design. J Comput Educ. (2021) 8:1–32. doi: 10.1007/s40692-020-00169-2

